# This is Jeopardy! A flexible coverage-based schedule model to address wellness for pathology training programs

**DOI:** 10.1016/j.acpath.2023.100087

**Published:** 2023-07-14

**Authors:** Jason V. Scapa, Bita V. Naini, Sheeja Pullarkat, Peggy S. Sullivan

**Affiliations:** Department of Pathology and Laboratory Medicine, David Geffen School of Medicine at the University of California, Los Angeles, CA, USA

**Keywords:** Graduate medical education, Paid time off, Resident curriculum, Resident schedule, Resident wellness

## Abstract

Scheduling rotations for a pathology training program involves balancing educational requirements, service coverage, and paid time off (PTO). Absences can affect training as residents cross-cover, managing multiple services at once. Other specialties utilize a “Jeopardy” based system for covering absences. In this system, residents on outpatient services are “jeopardized” to cover inpatient services for trainee absences. Borrowing this concept, we created a schedule model with a “Jeopardy-Elective” (JE) rotation to support resident absences. Prior to 2018–19, our residency program consisted of a 12 month-long rotation schedule. We adopted a 13 four-week block rotation model system, adding four JE rotations per resident over the course of training. The JE resident covered services during trainee absences and spent the remaining rotation on elective. We then conducted a pre- and post-intervention survey of all residents who trained in both systems. Following the change in schedule model, our results showed a statistically significant increase in resident satisfaction with taking PTO (p = 0.0014), finding coverage (p = 0.0006), and taking a sick day (p = 0.03). The mean number of days covered by the JE resident was 8.5 ± 2.7 workdays (out of 20). PTO usage increased from 16 to 20 days/resident while mean number of sick days decreased from 1.7 to 1.3 days per resident. There was overwhelming support with 82% of residents wanting to retain the new system going forward. Through use of the JE rotation, our program improved service coverage issues and resident satisfaction, with the long-term goal of enhanced resident well-being and enriched resident learning experiences.

## Introduction

There are three main principles that must be balanced when designing a residency program schedule: (1) education and curriculum requirements for training; (2) adequate service coverage of rotations reliant on trainees; and (3) allowing residents to take allotted paid time off (PTO)^1^^,^^2^^,^^3^^,^^4^. Once the schedule is constructed, it is often strained by absences that can come from many sources: PTO, sick days, family/medical leave, jury duty, etc. When an absence occurs, trainees from other services may be asked to cross-cover, which can leave them scrambling to manage multiple services at once. It is becoming more apparent that burnout is widespread throughout medicine, and pathology is not immune.^5^ With approximately 35% of pathology residents reporting symptoms of burnout, scheduling changes can contribute to reduced well-being if they increase the amount of work residents must perform to keep services operational.^6^ Additionally, there are educational considerations, since cross-covering two services may impact resident learning on their originally scheduled rotation.

The Accreditation Council for Graduate Medical Education (ACGME) has over the years addressed resident wellness and burnout through initiatives like duty hour requirements.^7^^,^^8^ In addition, the ACGME requires programs to allow for trainees to take care of personal health appointments, which often occur during business hours while they are on service.^9^ Even so, many physicians and residents continue to show up to work sick because of obligations to colleagues and patient care.^10^

To address the challenges created by trainee absences, many clinical programs employ a so-called “jeopardy” system. In this system, a resident on an outpatient clinic rotation or elective is called to cover an inpatient service when an unexpected resident absence occurs. This is possible for clinics that can operate without a trainee, while inpatient services rely on residents to run the day-to-day operations of a busy hospital service with more critically ill patients. To our knowledge, we are not aware of a pathology residency program that has published their experience of implementing a formalized jeopardy system before. Herein, we incorporate this concept in our residency scheduling model and describe its impact on resident satisfaction, PTO and sick day usage.

## Materials and methods

Our pathology training program is located at a large West Coast academic care center. The training program has four sites with the vast majority of training occurring at our main on-campus quaternary hospital and medical center. We have over 50 total clinical trainees comprised of 22 pathology residents [almost entirely anatomic and clinical pathology (AP/CP) combined track], approximately 20 A P fellows and 10 C P fellows (including clinical Ph.D. level fellows). For the pathology residency training program, we have an integrated AP/CP model with AP and CP rotations mixed throughout the four-year curriculum.

In 2018, our surgical pathology rotations were comprised of seven subspecialty-based services with each resident rotating through each subspecialty twice over training (14 total rotations). Residents were given 3 “true” electives over the course of their training. Regarding service coverage, our schedule included about 20 A P or CP rotations each month that relied heavily on a trainee (either resident or fellow) to support day-to-day operations of the service.

Prior to the academic year 2018–19, our program used a calendar month-based rotation model in which an AP/CP resident would have 48 rotations over their four-year curriculum (Prior System). Service transitions occurred on the first day of the new calendar month, regardless of the day of the week.

Residents did not have pre-scheduled PTO in the prior system. Residents were provided with 20 ‘flexible’ PTO days (four weeks) that could be applied to any weekday with the caveats that they could not use more than five days per rotation and must find their own service coverage, if needed. PTO requests required chief resident, service faculty, and program director approval. All personal health appointment requests were supported.

In the prior system, there was not a formal process for requesting additional trainee support (for example, in the case of an unexpected absence). Generally, the program and chief resident were notified and would explore various trainee-based solutions on a case-by-case basis (e.g., the chief resident would help out or seek trainee volunteers on ‘lighter’ services to help out).

For academic year 2018–19, a 13 four-week block model was proposed (New System). This allowed a four-year AP/CP resident to have 52 total rotations in training with service transitions occurring on Monday mornings every four weeks. With four extra rotations, a Jeopardy-Elective (JE) rotation was created that would occur four times over the course of training. Additionally, two separate assigned vacation weeks (ten workdays) were built into the schedule (or ‘pre-scheduled’) with coverage provided by the JE resident, if needed. Residents retained ten days of flexible PTO with the same caveats as in the prior system.

**Fig. 1:**
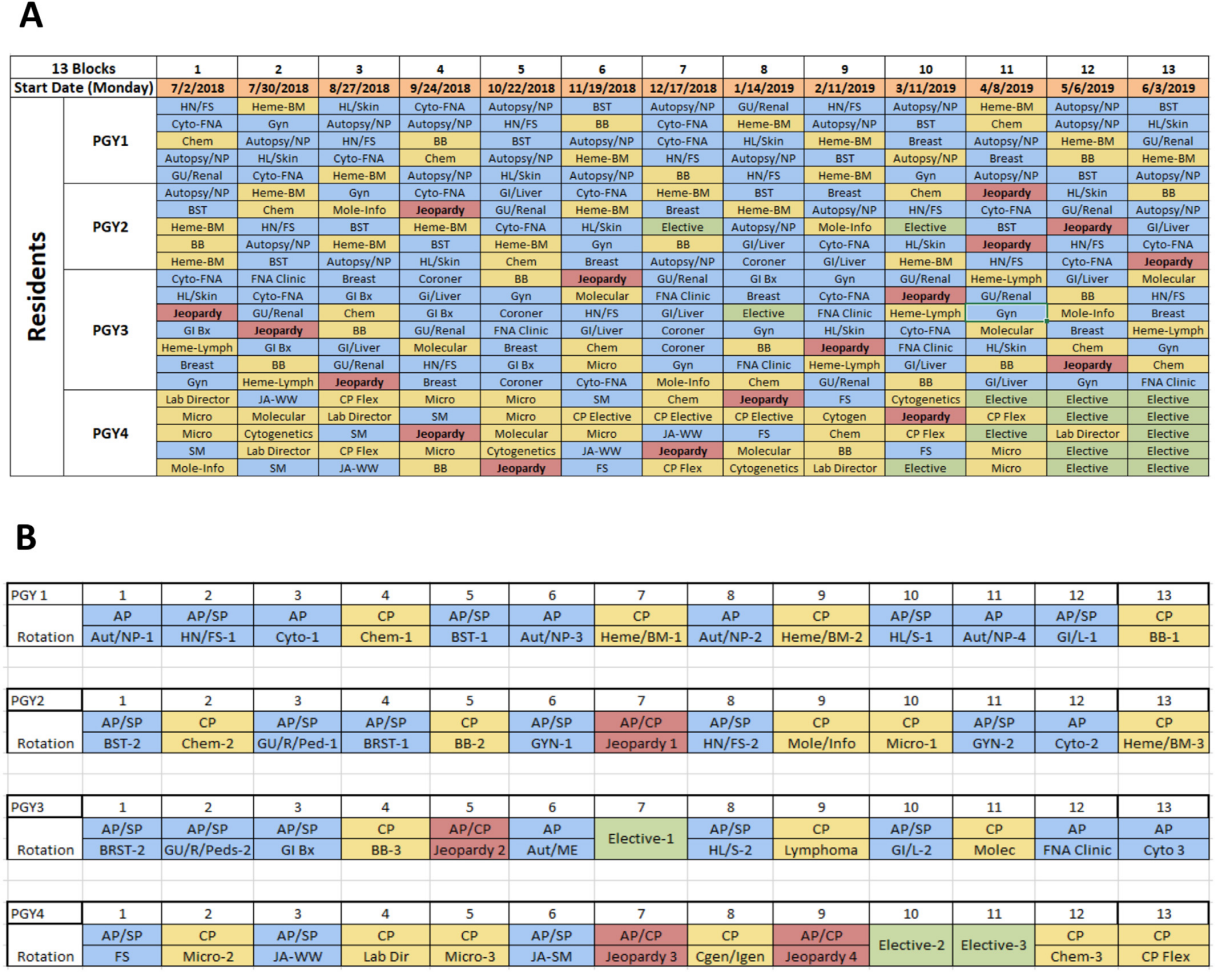
(A) Feasibility of the Proposed Jeopardy Based Schedule for Academic Year 2018–19. The figure shows a 13 four-week block schedule with a Monday rotation start date. All PGY2-4 residents complete one JE rotation (labeled in red color). At least one resident is on JE for all thirteen blocks. Blue color indicates AP rotations, gold color indicates CP rotations, and green is an elective. (B) The Proposed Schedule for a Single Prospective AP/CP Resident over a Four-Year Period. In the expanded 13-block schedule, four extra blocks are filled with a JE rotation in the PGY2-4 years of training, including two JE rotations the final year (highlighted in red). The mock four-year schedule for a single AP/CP resident satisfies all ACGME and department requirements for AP rotations (labeled in blue), CP rotations (labeled in gold), and three “true” elective rotations (green). Abbreviations: SP – Surgical Pathology, Aut – Autopsy, NP - Neuropathology, HN – Head & Neck Pathology, FS – Frozen Section, Cyto – Cytopathology, Chem – Clinical Chemistry, BST – Bone and Soft Tissue, Heme – Hematopathology, BM – Bone Marrow, HL – Heart/Lung Pathology, S – Skin or Dermatopathology, GI – Gastrointenstinal Pathology, L – Liver Pathology, BB – Blood Bank, GU – Genitourinary Pathology, R – Renal Pathology, Ped – Pediatric Pathology, BRST – Breast Pathology, GYN – Obstetrical and Gynecologic Pathology, Mole – Molecular Pathology, Info – Pathology Informatics, Micro – Clinical Microbiology, GI Bx – GI biopsies, ME or Coroner – Forensic Pathology, Molec – Molecular Pathology, FNA – Fine Needle Aspiration, JA-WW – Junior Attending in Surgical Pathology on the Westwood Campus, Lab Dir – Laboratory Director rotation in Clinical Pathology, JA-SM or SM - Junior Attending in Surgical Pathology on the Santa Monica Campus, Cgen or Cytogen – Cytogenetics, Igen - Immunogenetics, CP-Flex or CP Elective – Advanced Clinical Pathology Elective. (For interpretation of the references to color in this figure legend, the reader is referred to the Web version of this article.)

We performed a feasibility assessment to ensure every service that relied heavily on trainee support was able to be covered each block as well as making sure at least one resident was on JE per block to cover unexpected absences. We also needed to ensure that the JE resident could adequately cover scheduled PTO. Fig. 1A shows the proposed resident schedule for the 2018-19 academic year. Every post-graduate year (PGY) 2 through 4 resident was scheduled for one JE rotation. Because of the lack of service experience for several rotations, first-year residents were not scheduled on the JE rotation.

In addition, we sought to prove that an AP/CP resident could satisfy all their ACGME, board certification and department program requirements to graduate within four years. Fig. 1B shows the proposed schedule of a single prospective AP/CP resident over a 4-year period. With the expanded 52-block schedule, a single resident would complete four JE rotations during their PGY2-4 years of residency training, including two JE rotations their PGY4 year and retain all training requirements. After departmental approval, the program proceeded with the new schedule model.

The new system was implemented on July 1, 2018. During the year, the JE resident would notify the program director and scheduling chief resident of their JE “elective” preference prior to starting the JE rotation. In the event of an unexpected absence, the chief resident would notify the JE resident in the morning that they would cover the service for the day and therefore would not participate in their elective. On other weeks of the rotation, the JE resident may already be covering a resident on scheduled PTO. JE residents were also allowed to take flexible or scheduled PTO if not needed for planned coverage or a second JE resident was available to cover unexpected absences.

To assess resident opinions of the prior system and new system, we conducted an anonymous survey prior to the schedule model intervention (March 2018; Supplemental Material 1) and again seven months (8 blocks) into the intervention (February 2019; Supplemental Material 2). We performed reassessment early in order to determine whether to retain the model for the upcoming 2019–2020 year for schedule planning purposes. Student t-tests were performed to assess for statistically significant changes in satisfaction pre- and post-intervention. Where applicable, means were reported with the standard error at the 95% confidence interval. We also calculated average PTO and sick days taken per resident the year before and after the new system was implemented, based on department records. Averages were calculated including and excluding large segments (greater than three weeks) of continuous PTO taken due to significant personal/health circumstances.

## Results

### Assessment of the prior system

Twelve out of sixteen residents (75%) trained in both schedule systems and responded to both surveys. Additionally, all five of the new first year residents, who only trained in the new system model, responded to the February 2019 survey.

The pre-intervention survey asked residents to assess parameters on a five-point scale with 1 being “very unsatisfied” and 5 being “very satisfied”. Residents reported the lowest satisfaction with ease of finding coverage (2.36 ± 0.31) and ease of taking PTO (2.58 ± 0.38) in regards to the PTO request system. Prior to the switch, the most anticipated features of the new system included gaining extra electives in the form of the JE rotation and pre-scheduled PTO. In the prior system, residents reported using only 16.7 ± 1.7 PTO days of 20 eligible workdays. When asked about anticipated coverage days in the new system, a plurality of residents (46%) anticipated covering 5–10 days over the course of the 20-day rotation.

In addition, residents reported low satisfaction with ease of service transitions (2.33 ± 0.31) in regards to the prior system. Another highly anticipated feature of the new system was having the time to prepare service handoffs over the weekend.

### Satisfaction with new system

**Fig. 2:**
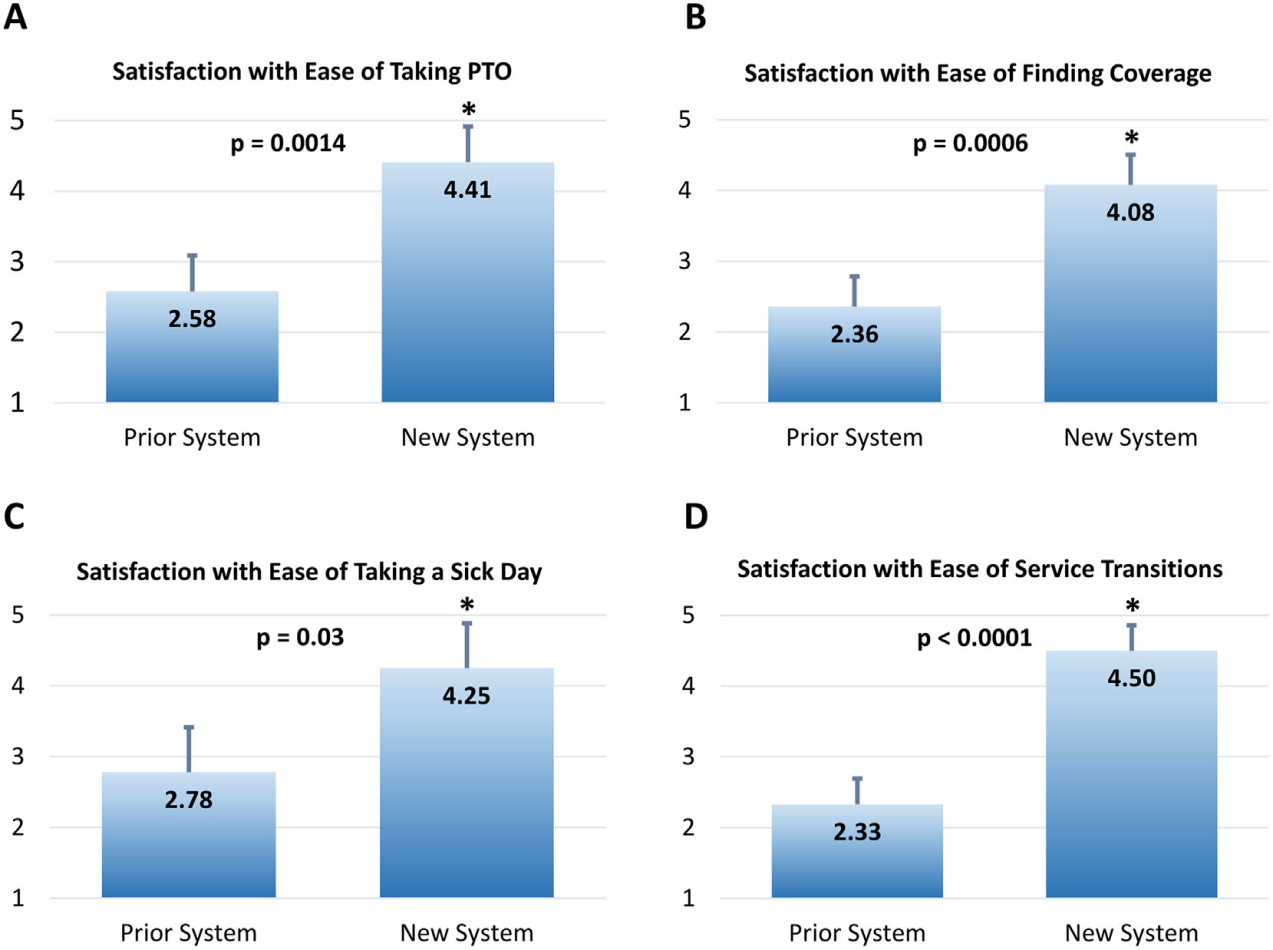
Resident Satisfaction with Schedule Change. Survey results demonstrate that residents had increased satisfaction in the ease of service transition (A), taking PTO (B), finding coverage (C), and taking a sick day (D) in the new system compared with the prior system. Satisfaction surveys were scored from 1 to 5 with 1 being very unsatisfied and 5 being very satisfied. P-values display results from unpaired two tailed *t*-test at 95% confidence interval. Error bars show standard error. Asterisks denote statistical significance at 95% confidence interval.

**Fig. 3:**
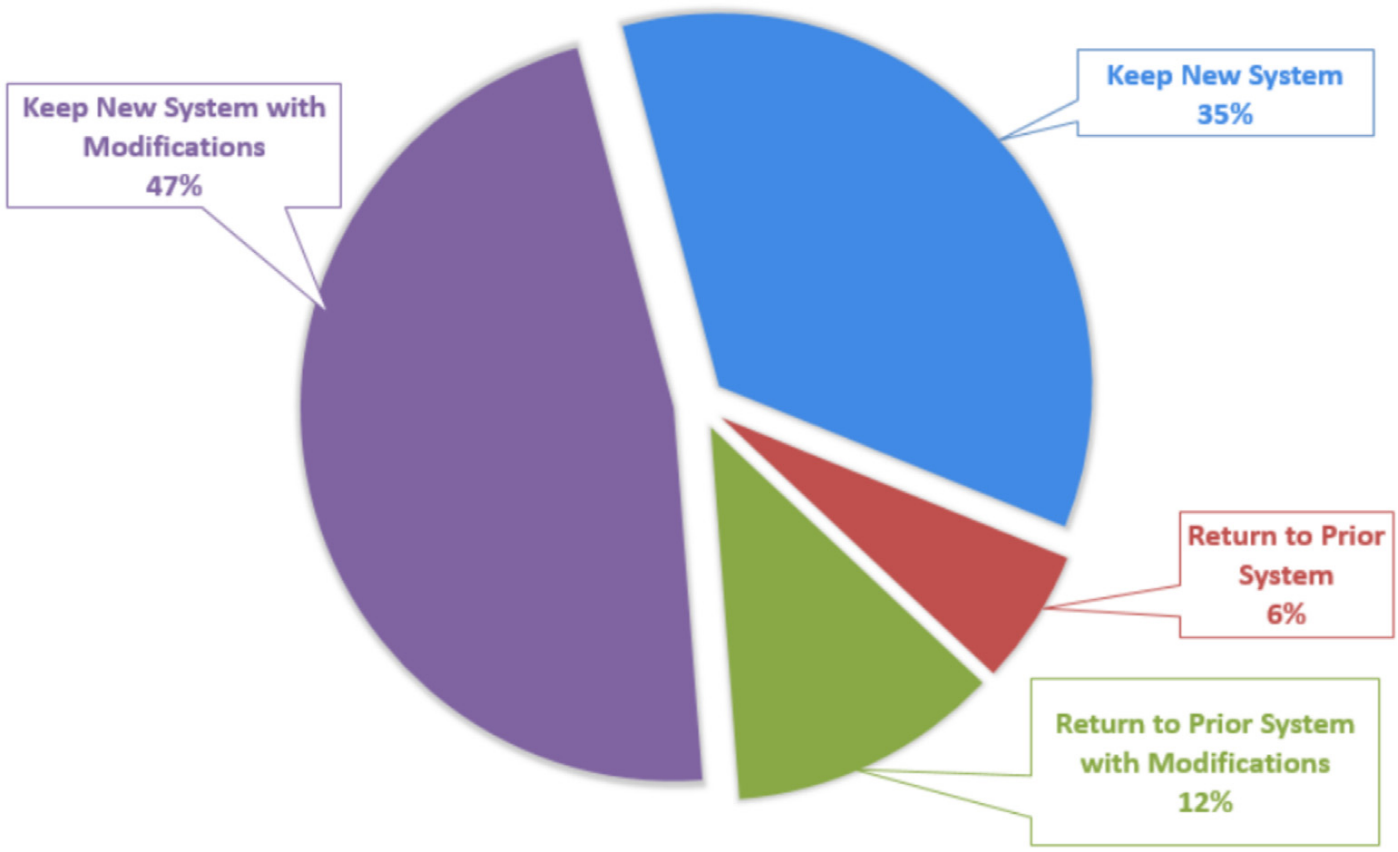
Overall Opinions on Maintaining Jeopardy-Based System for the Subsequent 2019–2020. A large majority of residents are in favor of keeping the New System for the next academic year (82%). Comments for modifications centered on further clarifying instances where Jeopardy should be called and to prevent overutilization by busy services.

Seven months (eight blocks) post-intervention data using the same five-point scale showed statistically significant improvement in satisfaction in a number of parameters: ease of taking PTO (4.41 ± 0.34, p = 0.0014), ease of finding coverage (4.08 ± 0.56, p < 0.001), and ease of taking a sick day (4.25 ± 0.46, p = 0.03) (Fig. 2). In addition, ease of service transitions (4.5 ± 0.19, p < 0.001) also showed statistically significant improvement. Residents who had previously rotated on the JE rotation at the time of the post-intervention survey (n = 7) reported covering an average of 8.5 ± 2.7 days (out of 20 workdays). The most popular chosen elective component of the JE were the gastrointestinal biopsy service, dermatopathology service, and lymphoma/solid tissue hematopathology service. When asked whether to continue the new system into academic year 2019–2020 and beyond, the resident cohort (including first-year residents) responded with 47% wanting to keep the new system with some modifications, 35% wanting to keep the new system as is, and a combined 18% wanting to return to the prior system with or without modification (Fig. 3). The highest rated aspects of the new system were the pre-scheduled vacations and rotation switches occurring over the weekend (data not shown).

All respondents were given a free text option to provide suggestions and comments. All seven residents who provided comments called for clarifying the instances when the JE resident should be pulled off to cover or wanted to prevent JE overutilization by services that had a higher case volume. These suggestions came from four residents who wanted to keep the new system with modifications and three residents who wanted to return to the prior system, with or without modifications. The two residents who wanted to return to the prior system with modifications cited the inability to have a meaningful elective experience while covering other trainees during the JE rotation. One of these residents reported the perception of residents taking an “exponentially higher” number of sick days in the new system. Finally, one resident who wanted to keep the prior system without modifications felt the new system increased schedule complexity and that the new system did not improve the ease of finding coverage or taking PTO enough to retain going forward.

At the end of the academic year, we examined the average PTO and sick days in the prior system and new system. The average number of PTO days taken by a resident increased from 16.4 days in the prior system to 20.0 days (using all eligible PTO) per resident in the new system. When large segments of PTO (greater than 3 weeks) taken due to significant personal/health circumstances were added to the average calculation, the prior system PTO average increased to 19.2 days while the new system PTO average remained unchanged. The average number of sick days taken by a resident decreased from 1.7 days to 1.3 days per resident in the new system (Table 1).

**Table 1:** Comparison of Average Paid Time Off (PTO) and Sick Days Taken by Residents in the Prior System and New System. All residents take their full allotted PTO in the new system. Average sick days taken per resident is slightly decreased in the new system.

	Prior System	New System
# PTO days (avg/resident)	16.4^a^	20.0
# Sick days (avg/resident)	1.7	1.3

a Average calculated by excluding large segments of PTO take due to unusual personal/health circumstances.

## Discussion

Here we report an educational improvement system in pathology training that emphasizes coverage-based principles through the use of a JE rotation, created by transitioning to a 4-week block schedule. We have shown that this new system has increased resident satisfaction in ease of taking PTO and ease of taking a sick day while retaining ACGME/program requirements and preserving operational needs. We have shown that this system also increased resident satisfaction in service handoffs. Overwhelmingly, 82% of residents wanted to keep the new system with a plurality requesting some modifications. The comments centered around further clarifying appropriate jeopardy usage and preventing overutilization by services that needed more residents for the amount of work, not because a trainee was absent.

Resident well-being is a large focus in medical education.^11^ Pathology programs are actively developing wellness strategies that specifically address well-being within pathology training.^12^ While duty hour limits have helped in some respect, resident burnout exacerbated by the pandemic has made resident wellness in training more important than ever.^13^ A recent nationwide study of general surgery residencies reports an association of resident wellness with flexibility in scheduling and perception of program responsiveness.^14^ This includes flexibility in vacation scheduling, time to safely transition patient care, and flexibility in educational experiences (e.g., electives). In addition, the study reported that “residents who felt that their co-residents would willingly step in when a resident needs flexibility were three times more likely to report program responsiveness”.

Creating flexible systems that encourage full use of PTO, encourage ease of taking a sick day, customize educational experiences, and reduce burnout should be an emphasis going forward. A “flexible” PTO system without pre-scheduled PTO days provides a trainee with the ability to take time off in customized increments as needed. However, programs may not be aware of “barriers” that may impact full use of PTO or taking a sick day in these “flexible” systems, especially when policies include placing the burden of finding coverage on the requesting resident. Some barriers may include the reluctance to ask a co-resident to cross-cover a busy service or difficulty finding a resident willing or able to cross-cover, especially in situations of prolonged co-worker absences due to significant personal/health circumstances. Other issues may include the resident motivation to appear “present,” “engaged,” and “hard-working” to ensure positive evaluations or strong recommendation letters for future fellowship/job prospects. In addition, residents dedicated to the care of their patients may simply not recognize their own need for rest. It is important for programs to recognize these influences and to cultivate a culture of taking PTO or a sick day as an important part of one's professional responsibility as a physician.

Additionally, ACGME requirements mandate programs allow residents time during work hours to attend personal care appointments, which can be difficult in a traditional schedule model.

Our jeopardy system could help achieve this requirement since there is at least one resident who can cover for the few hours necessary for another trainee to attend to their personal care appointments. Having a structured coverage system where all residents “pitch in” contributes to a workplace culture of supporting PTO, shared responsibility, and teamwork.

From an educational standpoint, our model also allowed time for additional exploration of subspecialties within pathology. With the pressure to choose a fellowship subspecialty early in training, it is often difficult for trainees to rotate through all the subspecialties of pathology before making a decision on which to pursue for fellowship. While residents retained three “true” electives in the new system, the JE rotation may provide trainees with additional time to explore fields in pathology they may not get exposed to early in training. For example, solid tissue hematopathology and mucosal gastrointestinal biopsy service have both been traditionally an upper year rotation at our training program. The JE rotation permitted several second and third year residents the chance to see solid tissue hematopathology and gastrointestinal biopsy cases earlier in their training, allowing them to make a more informed decision on whether to pursue a fellowship in those fields.

Finally, by converting to a 13 four-week block model, we helped address safe patient handoffs through the residency schedule. Patient safety should always be a high priority during service transitions or patient handoffs. It has been shown that incomplete handoffs that occur between shift or rotation switches are a large source of clinical error.^15^^,^^16^ Even in pathology, there have been creative solutions to minimize miscommunications and ineffective handoffs.^17^ Our previous 12-month rotation model had the potential for rotation switches and handoffs to occur any day of the work week, causing residents to juggle both giving and receiving patient cases “on the fly” the morning of the first day of the month. In some instances, residents would need to complete work from their previous rotation, such as grossing a specimen fixed overnight or previewing slides, in the first few days of the month while they begin rotations on other services. In the new system, rotation switches occur only on Mondays, allowing time to finish case preview or grossing, review the pending cases or patient list, and perform a thorough written service handoff to the incoming trainee over the weekend prior to the rotation switch. The post-intervention survey data showed that residents had a higher satisfaction rate in service handoffs.

While we sought to optimize the schedule to address resident well-being, education, and patient handoffs, there are limitations to our proposed model. There were instances when there was an unexpected absence while the JE resident was also absent on their PTO. In these situations there was some traditional cross-covering (using the prior system) that did occur. While we had considered scheduling all four weeks of PTO at the beginning of the year, this option was met with strong opposition from residents. Thus, we retained some flexible PTO. In order to help improve ease of taking PTO and uninterrupted learning on core (non-JE) rotations, we also scheduled PTO during the JE rotation and supported occasional flexible PTO requests. This was generally (but not always) done when a second JE resident was present. By doing so, we created additional complexity in managing JE requests.

In addition, survey comments from the residents point to apprehension about “requests” for the JE resident to help on busy services that technically have sufficient coverage to begin with. Our data show that on average a JE resident covered 8.5 days or one and a half weeks for a 4 week rotation. A surgical residency program with a similar JE rotation reports a lower number of days of coverage (2.6 days for a 4–7.5 week rotation).^18^ Deciding when a resident should be “jeopardized” to fill in was difficult during our inaugural year, with a lack of consensus from the service attending, program director, chief resident, and the JE resident.

We did not formally track the number or type of JE resident requests (e.g., for an unexpected absence versus a “busy” service). The requests however came from multiple services and multiple individuals (resident, fellow, faculty, staff) for varying reasons including an unexpected resident absence, unexpectedly high case volume, unexpected fellow absence, inexperienced resident, or a combination of these situations. We did not anticipate this need and handled these requests on a case-by-case basis.

Interestingly, our JE rotation and the types of JE requests that we received generated multiple discussions on rotation/program improvement and the delineation between operational need and educational requirement. Resident-led rotation/workflow improvement projects were generated. Service areas needing greater operational support beyond trainee educational requirements were directed to department/operational leadership to help identify and establish additional non-trainee resources. The pandemic (and its impact on our operations and workforce) further clarified the delineation between operational need and educational requirement in our department. With support from our department Chair, our program underwent a major curriculum revision, implemented in 2022. We continue to retain the JE resident coverage model for unexpected absences. For various reasons beyond the scope of the manuscript, we now have four weeks pre-scheduled PTO and do not allow PTO during JE coverage. With improved understanding of appropriate JE case use, we adhere to a strict program director-controlled gatekeeper model for JE utilization.

In addition to the potential for JE overutilization, there are some “down-the-road” caveats one should be mindful of in applying our schedule model to a calendar-based residency program. By switching to a four-week block model, there are now 13 rotations in the PGY1 year instead of 12 rotations in a calendar-month year. Since residents have a set number of required rotations, they will complete their rotations earlier, which will affect the resident experience over time. Re-balancing rotation length and/or frequency may be necessary in subsequent years. There will also be slightly less resident coverage of clinical services overall with at least 1 resident “away” on the new JE rotation during every block. While anecdotally, the impact was minimal in our first year, it is important the program monitors the transition to this model, especially on busier rotations. Working closely with the department to make minor program or rotation adjustments, and monitoring changes (using some of the methods described here) are a necessary part of any program intervention and will help to preserve the aims of the JE coverage model.

Programs have infinite ways to schedule residents in a training program to accomplish their education goals and coverage needs. No matter what schedule system a pathology training program chooses to implement, creative and flexible schedule models – such as the one we propose here – that address resident well-being will be critical for all pathology residency programs.

## Declaration of competing interest

The Author(s) declare(s) that there are no competing interests.
