# Supplementary material for: This is Jeopardy! A flexible coverage-based schedule model to address wellness for pathology training programs
Source: Acad Pathol. 2023 Jul 14;10(3):100087. doi: 10.1016/j.acpath.2023.100087 (PMC10371805; doi:10.1016/j.acpath.2023.100087)
Supplement: Multimedia component 1 [file mmc1.pdf]

Supplemental Material 1

Pre-Intervention Survey

1. Please score your satisfaction of the following aspects of the "old system" (1-very unsatisfied to 5- very satisfied). "Old System" refers to the rotation schedule based on 12 months with 20 vacation days and no jeopardy coverage.

	1	2	3	4	5	N/A
Ease of transitions from rotation to rotation at end of each month						
Ease of taking vacation						
Ease of finding coverage						
Ease of getting rotations you wanted						
The amount of call you took						
Doing 12 rotations a year						
Ease of taking a sick day						
Ability to attend national/regional conferences						

2. Please score your expectations of the "new system" improving the following aspects of the "old system". "New System" refers to the rotation schedule based on 13 months with 2 weeks scheduled vacation, 10 ex days, and a jeopardy coverage rotation.

	1- I expect it to be really worse than "old system"	2- I expect it being some worse off than "old system"	3- I expect no worse or better than "old system"	4- I expect some improvement	5- I expect major improvement	N/A
**Ease of transitions from rotation to rotation at end of each month**	⬭	⬭	⬭	⬭	⬭	⬭
**Ease of taking vacation**	⬭	⬭	⬭	⬭	⬭	⬭
**Ease of finding coverage**	⬭	⬭	⬭	⬭	⬭	⬭
**Ease of getting rotations you wanted**	⬭	⬭	⬭	⬭	⬭	⬭
**The amount of call you took**	⬭	⬭	⬭	⬭	⬭	⬭
**Ease of taking a sick day**	⬭	⬭	⬭	⬭	⬭	⬭
**Ability to attend national/regional conferences**	⬭	⬭	⬭	⬭	⬭	⬭

3. Please rank all the following aspects in terms of what you are most looking forward to in the "new system". (1 = Aspect most looking forward to)

	1	2	3	4	5
**Scheduled vacation**	⬭	⬭	⬭	⬭	⬭
**13 blocks**	⬭	⬭	⬭	⬭	⬭
**Coverage by the Jeopardy system**	⬭	⬭	⬭	⬭	⬭
**Rotation switches occurring over the weekend**	⬭	⬭	⬭	⬭	⬭
**Extra yearly elective as part of Jeopardy rotation**	⬭	⬭	⬭	⬭	⬭

4. What are your pre-schedule change thoughts on the Jeopardy coverage system? (1-I really don't like this idea to 7- I really like this idea)

I really don't like the idea

1 ◯

2 ◯

3 ◯

4 ◯

5 ◯

6 ◯

7 ◯

I really like the idea

5. What are your pre-schedule change thoughts on the 2 weeks scheduled vacation? (1-I really don't like this idea to 7- I really like this idea)

I really don't like the idea

1 ◯

2 ◯

3 ◯

4 ◯

5 ◯

6 ◯

7 ◯

I really like the idea

6. On average, how many flex vacation days were you able to use a year in the "old system"? Please put a number 1 to 20. 20 means you used all your vacation days on average every year. You can extrapolate for the rest of 2017-18.

______

7. In a 20-workday month when you are on jeopardy-elective, how many days do you think you will be pulled off of your elective portion to help cover?

- None
- 1-2 days
- 3-5 days
- 5-10 days
- 10-15 days
- 15-20 days

8. Are there any free text comments you would like to state about the changes in scheduling systems?

______
