# Supplementary material for: This is Jeopardy! A flexible coverage-based schedule model to address wellness for pathology training programs
Source: Acad Pathol. 2023 Jul 14;10(3):100087. doi: 10.1016/j.acpath.2023.100087 (PMC10371805; doi:10.1016/j.acpath.2023.100087)
Supplement: Multimedia component 2 [file mmc2.pdf]

Supplemental Material 2

Post-Intervention Survey

1. Post-graduate year for 2018-2019?

- PGY1
- PGY2
- PGY3
- PGY4

2. Have you been on the Jeopardy Rotation?

- Yes
- No

3. Please score your Opinions of the "new system" improving the following aspects of the "old system". "New System" refers to the rotation schedule based on 13 months with 2 weeks scheduled vacation, 10 flex days, and a jeopardy coverage rotation.

	1- This is worse than "old system"	2- This is somewhat worse than "old system"	3- No change between "old system"	4- This is somewhat better than "old system"	5- This is better than "old system"	I'm a PGY1 and did not experience "old system"	N/A
**Ease of transitions from rotation to rotation at end of each month**	◯	◯	◯	◯	◯	◯	◯
**Ease of taking vacation**	◯	◯	◯	◯	◯	◯	◯
**Ease of finding coverage**	◯	◯	◯	◯	◯	◯	◯
**Ease of getting rotations you wanted**	◯	◯	◯	◯	◯	◯	◯
**The amount of call you took**	◯	◯	◯	◯	◯	◯	◯
**Ease of taking a sick day**	◯	◯	◯	◯	◯	◯	◯
**Ability to attend national/regional conferences**	◯	◯	◯	◯	◯	◯	◯
**Overall resident well-being from a scheduling perspective**	◯	◯	◯	◯	◯	◯	◯

4. Please rank all the following aspects in terms of what are the best parts in the "new system". (1 = Aspect I like the most, 5 = least)

	1	2	3	4	5
**Scheduled vacation**					
**13 blocks**					
**Coverage by the Jeopardy system**					
**Rotation switches occurring over the weekend**					
**Extra yearly elective as part of Jeopardy rotation**					

5. What are your thoughts on the Jeopardy coverage system after implementation for 7 months? (1-I really don't like this idea to 7- I really like this idea)

I really don't like the idea

1
2
3
4
5
6
7

I really like the idea

6. What are your thoughts on the 2 weeks scheduled vacation? (1-I really don’t like this idea to 7- I really like this idea)

I really don't like the idea

1 ⬭
2 ⬭
3 ⬭
4 ⬭
5 ⬭
6 ⬭
7 ⬭

I really like the idea

7. For those who have done Jeopardy rotation, how many days did you physically cover another service? Please put a number 0 through 20. If you have not done it, please put 0.

____

8. Overall what are your thoughts on the new Jeopardy schedule system?

- I like the new system as is
- I like the new system with some modifications
- I would like to go back to the "old system" with some modifications
- I would like to go back to the "old system" the way it was

9. What are your thoughts about jeopardy covering flex vacation day(s) or absence due to a national meeting?

- [ ] I prefer the ability to ask Jeopardy resident to cover for flex days/conference. I also feel comfortable covering requests when on Jeopardy.
- [ ] I would prefer Jeopardy resident does not cover flex days/conference

10. Please state any ideas you have about how to modify the Jeopardy rotation specifically.

---

11. Please state any ideas you have about how to modify the whole new system.

---

12. Are there any other free text comments you would like to state about the change we implemented this year?
